# Illustrated histopathological features of fatal dengue cases in Colombia

**DOI:** 10.7705/biomedica.5016

**Published:** 2020-06-30

**Authors:** Jorge Alonso Rivera, Aura Caterine Rengifo, Édgar Alberto Parra, Jaime E. Castellanos, María Leonor Caldas

**Affiliations:** 1 Grupo de Morfología Celular, Dirección de Investigación en Salud Pública, Instituto Nacional de Salud, Bogotá, D.C., Colombia Instituto Nacional de Salud BogotáD.C Colombia; 2 Grupo de Patología, Dirección de Redes en Salud Pública, Instituto Nacional de Salud, Bogotá, D.C., Colombia Instituto Nacional de Salud BogotáD.C. Colombia; 3 Facultad de Odontología, Universidad Nacional de Colombia, Bogotá, D.C., Colombia Universidad Nacional de Colombia Universidad Nacional de Colombia BogotáD.C Colombia; 4 Subdirección de Innovación en Salud Pública, Dirección de Investigación en Salud Pública, Instituto Nacional de Salud, Bogotá, D.C., Colombia Instituto Nacional de Salud BogotáD.C. Colombia

Dengue is the most important arboviral disease in humans in tropical and subtropical countries and it is considered endemic by the World Health Organization. It has been estimated that 390 million cases occur annually, 96 of which are clinically diagnosed as symptomatic dengue fever ^1^. Although most infections are asymptomatic, the more severe forms of dengue (formerly called hemorrhagic or shock syndrome) may result in organ failure or death ^2^ with around a half-million cases reported each year, an estimated average of 10,000 fatal cases per year (between 1990 and 2013), and a peak in 2010 (11,302 fatal cases) ^2^. Dengue incidence has seen a 30-fold increase in the last 50 years ^3^ making it a major public health concern currently.

In Colombia, dengue virus infection has an endemic and epidemic behavior with a steady increment in the last 20 years. The Colombian national surveillance system reported the highest historic peak of dengue cases in 2010 (157,000 cases) with 9.777 severe cases and a worrisome number of 217 fatalities (2.28% lethality rate) ^4^. During 2011, there was a decrease in dengue cases but the lethality rate increased to 3.75% ^5^. The burden of the 2010 dengue epidemic was 14-fold higher than that of 2011 or 2012 (57.017 vs. 3.989 disability-adjusted life years were lost, respectively). Additionally, the estimation of the 2010 epidemic costs rose to USD$ 65.5 million, almost fourfold higher compared to a regular endemic or epidemic year. Approximately 30% of these costs was linked to loss of income due to fatalities.

Dengue is caused by a virus from the *Flavivirus* genus of the Flaviviridae family and presents four antigenically different serotypes (DENV-1 to 4). Each serotype can produce asymptomatic infections or clinical signs and symptoms ranging from a mild febrile disease to a severe infection characterized by the imbalance of endothelial function leading to massive plasma leakage, severe hemorrhage, and multi-organ failure ^6^. The infection can be fatal and involve organs such as the liver, brain, spleen, lungs, and kidneys ^7^.

Dengue virus can infect different cell types and its pathological manifestations are variable ^8^^,^^9^. In the most severe cases, the damage in the vascular endothelium results in plasma extravasation and hemorrhage, liver function impairment with high transaminase levels, and histologic alterations ^10^^-^^12^.

The histopathological analyses of fatal cases indicate that the liver and spleen are the most affected organs during dengue virus infection. In the liver, it is common to find small necrotic foci, microvesicular steatosis, hyperplasia and apoptosis of Kupffer cells, lymphocyte infiltration in the portal tract and Councilman bodies (necrotic foci, acidophilic bodies, and pyknotic nuclei) ^13^^-^^16^, although intranuclear glycogen can be occasionally found ^17^.

The histopathological analysis of the spleen usually shows interstitial edema and white pulp vascular and cellular congestion with reactive hyperplasia ^13^^,^^18^. Atypical alterations have also been reported in the kidneys, lungs, heart, and the brain with hemorrhage, edema, and leukocyte infiltrates but no specific morphological findings in each tissue ^8^^,^^19^^-^^22^. On the other hand, immunohistochemistry for DENV antigens has revealed different distribution patterns: from the location of antigens in a single organ per case to the presence of antigens in multiple organs ^8^^,^^9^^,^^18^^,^^22^^,^^23^.

In the present study, we aimed to describe, illustrate, and compare different histological alterations found in 95 fatal confirmed dengue cases using 87, 42, 32, 37, 22, and 16 samples of liver, spleen, kidney, lung, heart, and brain, respectively. We reviewed histopathological slides with hematoxylin and eosin staining from 95 cases belonging to the pathology archive at the Colombian *Instituto Nacional de Salud*. The confirmation of death due to dengue virus infection was done using molecular techniques and the analysis of the clinical history of the cases.

The main alterations found in the liver were necrosis (78.2%) and hyperplasia of Kupffer cells (82.7%) while in the spleen, reactive plasmacytosis (69%) and vascular congestion (92.9%) were the most frequent findings. Edema was the most common alteration in the lungs (83.8%) and the brain (68.8%). Interestingly, most heart (77.3%) and kidney (65.3%) tissues had a normal histopathological aspect without any other specific finding (figures 1-12). Figure 13 illustrates in more detail the frequencies of the alterations found in each tissue.

## The organs and its histopathological alterations

### The liver

**Figure 1:**
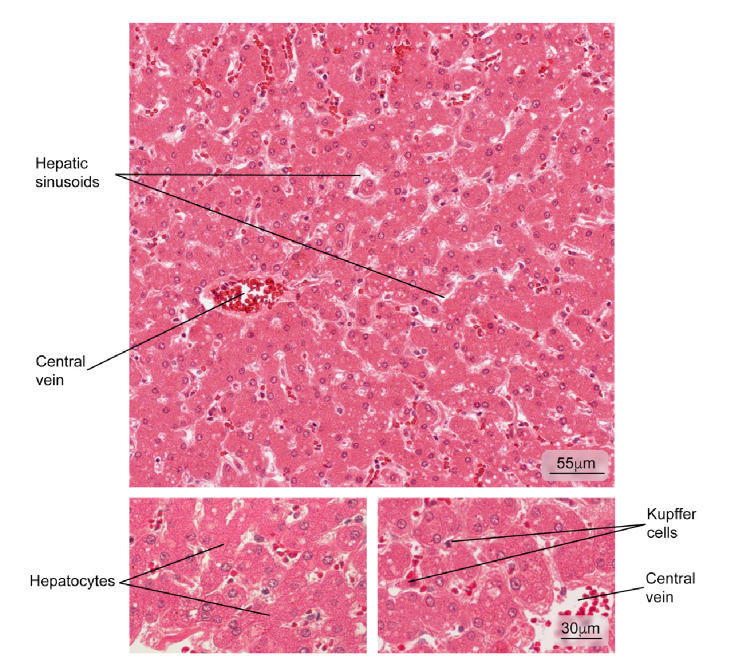
Normal liver tissue. Part of a hepatic lobule is observed; note the radial distribution of the hepatocyte plaques from the central vein. Some phagocytic cells (Kupffer cells) and hepatocytes in greater magnification are observed. Hematoxylin and eosin stain.

**Figure 2:**
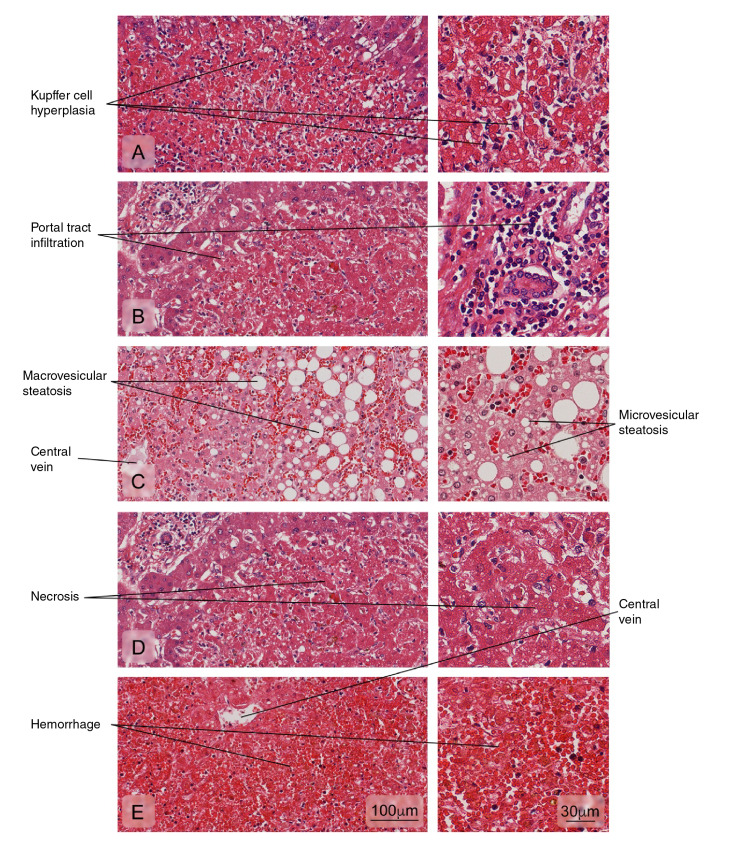
Alterations of the liver tissue showing: **A)** Kupffer cell hyperplasia; **B)** Portal tract leukocyte infiltration; **C)** Hepatic fatty degeneration (macro and microvesicular steatosis); **D)** An area of necrosis with loss of radial arrangement of hepatocyte plaques, a pale eosinophilic stain, and pyknotic or absent nuclei; **E)** Hemorrhages. Hematoxylin and eosin stain.

**Figure 3:**
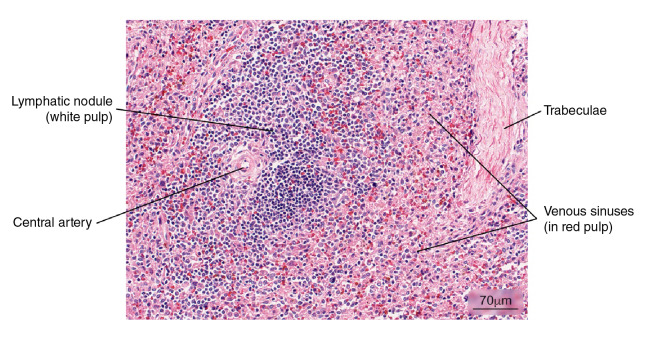
Normal splenic tissue. A small lymph node with a peripherally located central artery is observed. Connective tissue trabeculae are evident. Hematoxylin and eosin stain.

**Figure 4:**
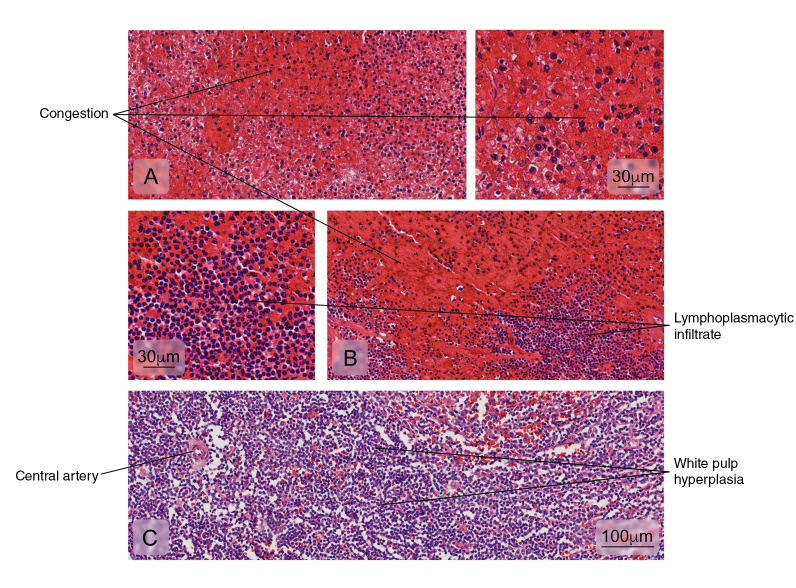
Different kind of splenic tissue alterations are illustrated in: **A)** Venous sinuses congestion in the red pulp, **B)** Lymphoplasmacytic cell infiltration in the red pulp, and **C)** White pulp hyperplasia. Hematoxylin and eosin stain.

### The kidney

**Figure 5:**
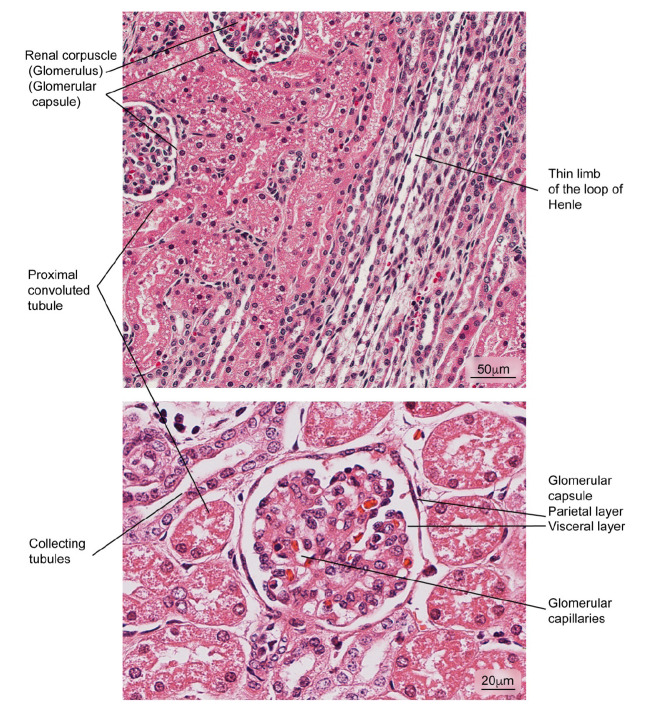
Aspect of normal kidney tissue. Renal corpuscles are observed in medullary areas and renal cortex; the glomerular capsule is shown in detail. Hematoxylin and eosin stain.

**Figure 6:**
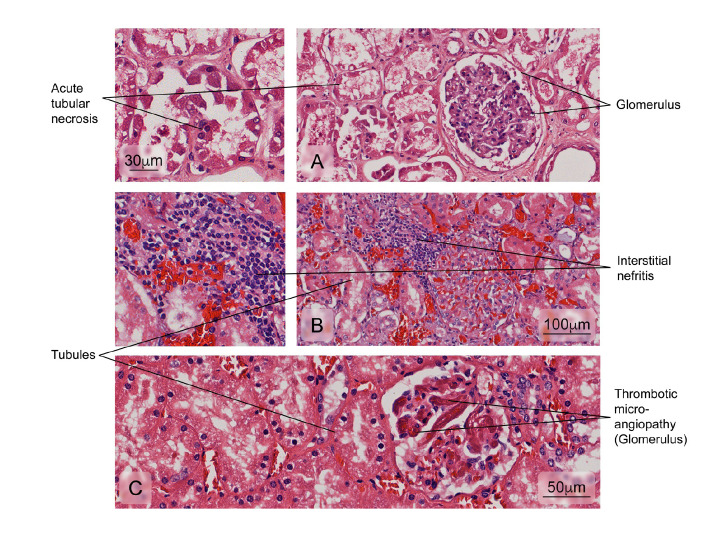
Alterations observed in renal tissue of cases. **A)** Necrosis of the tubular epithelium; **B)** Leukocyte infiltration of the interstitial space surrounding the renal tubules (interstitial nephritis), and **C)** Vascular lesions accompanied by intraluminal platelet thrombosis obstructing the vascular lumen (thrombotic microangiopathy). Hematoxylin and eosin stain. The heart

### The heart

**Figure 7:**
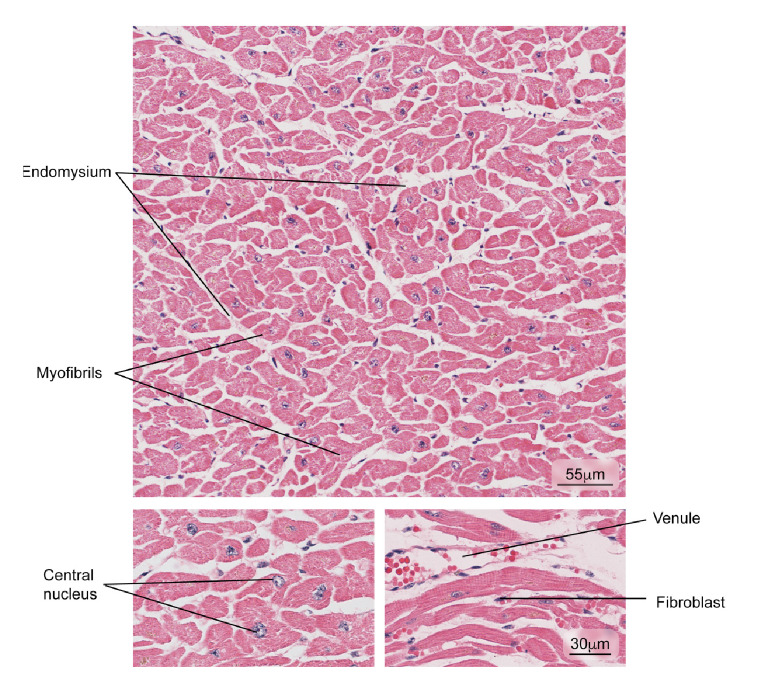
Aspect observed in patients with normal cardiac muscle tissue. The myocardium composed of cardiac muscle fibers, venules, and fibroblasts in the endomysium is observed. Hematoxylin and eosin stain.

**Figure 8:**
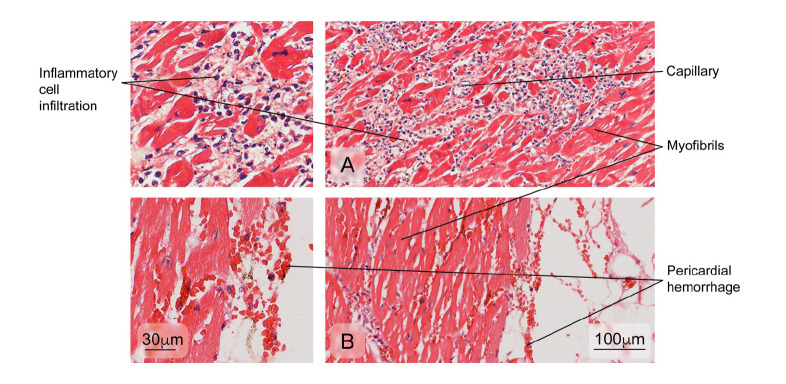
Aspect of cardiac muscle tissue in dead patients with anomalies. **A)** Inflammatory cell infiltration of the myocardium and **B)** pericardial hemorrhage. Hematoxylin and eosin stain.

### The lung

**Figure 9:**
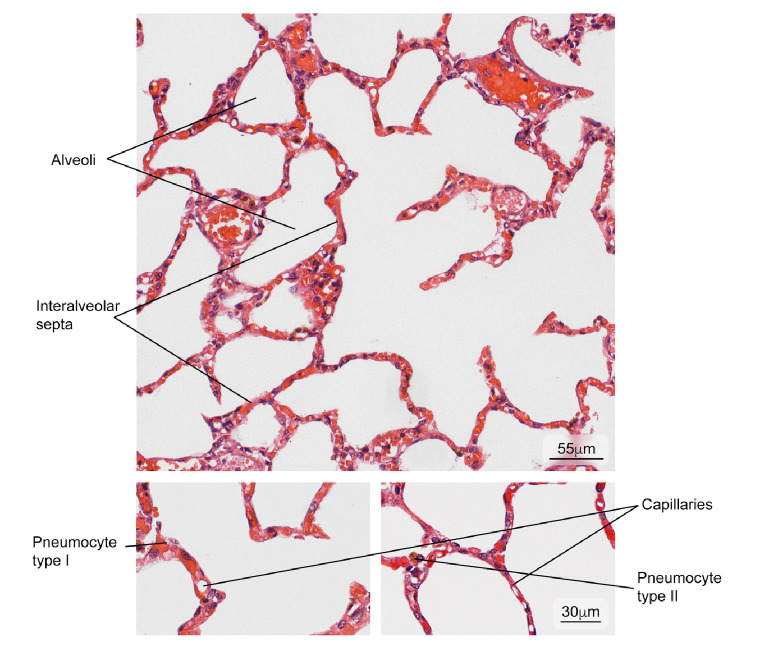
Normal lung tissue. Intrapulmonary structure with gaseous exchange (alveoli) and main cell types in the interalveolar septum (type I and II pneumocytes). Hematoxylin and eosin stain.

**Figure 10:**
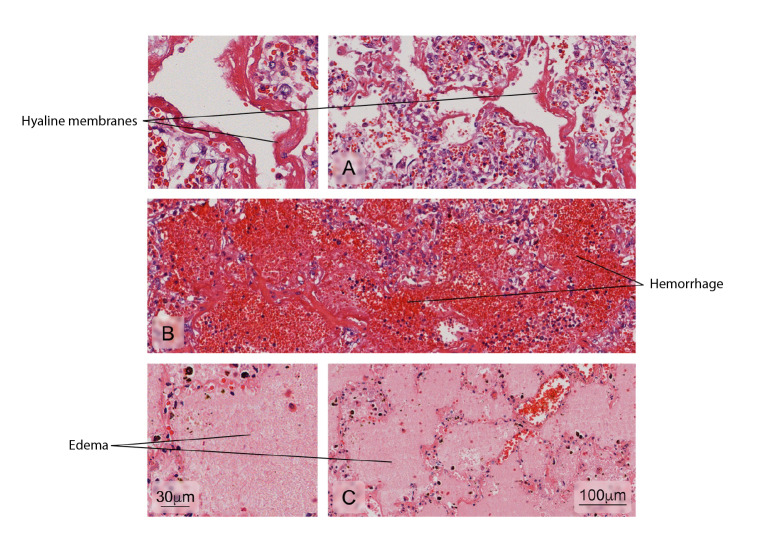
Alterations found in lung tissue of fatal dengue cases. Abnormal findings: **A)** Formation of hyaline membranes characteristic of diffuse alveolar damage (DAD); **B)** Severe hemorrhages in the alveoli; **C)** Presence of serous fluid in alveolar areas (edema). Hematoxylin and eosin stain

### The brain 

**Figure 11:**
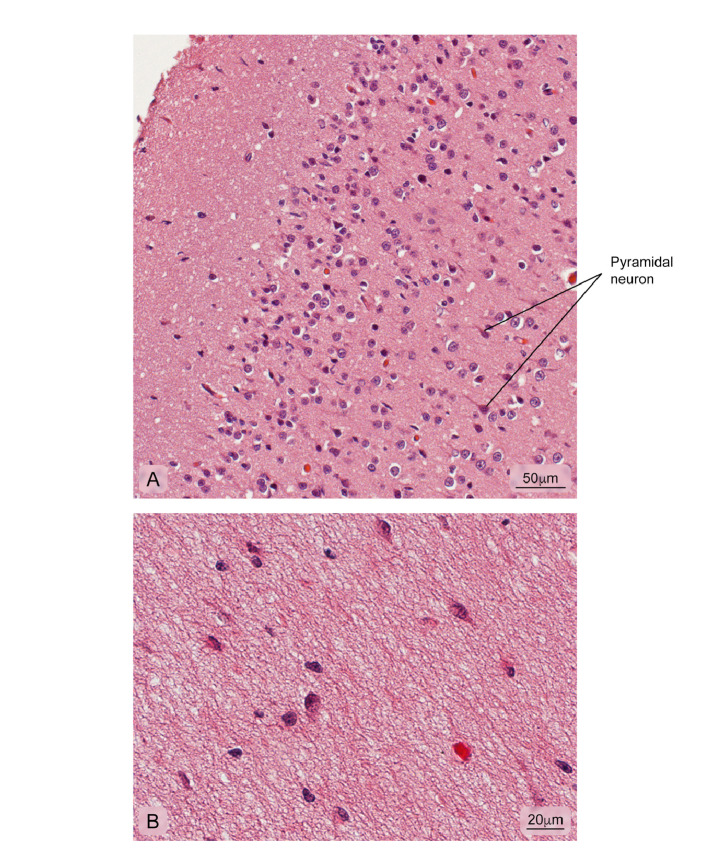
Normal cerebral cortex. **A)** Cerebral cortex outermost layer showing different kinds of neuronal bodies; **B)** More internal part of the cortex where the integrity of the neuropil is observed. Hematoxylin and eosin stain.

**Figure 12:**
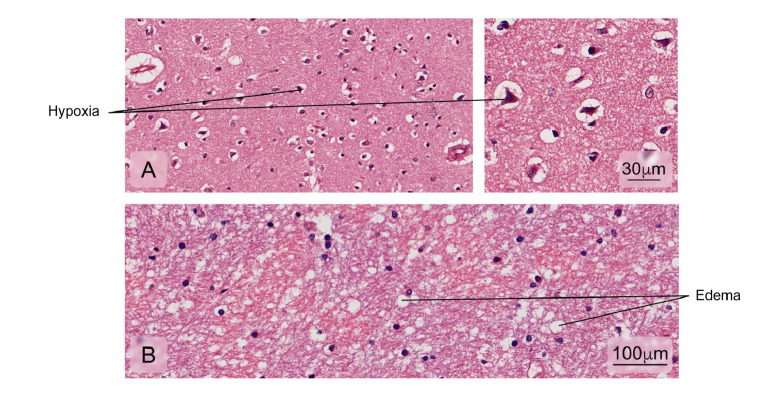
Alterations found in brain tissue-cerebral cortex. Some of the fatal cases presented:**A)** Decreased neuronal size due to retraction of the cytoplasm with nuclei pyknosis and hyperchromasia associated with hypoxic cortical changes; **B)** Edema. Hematoxylin and eosin stain.

**Figure 13:**
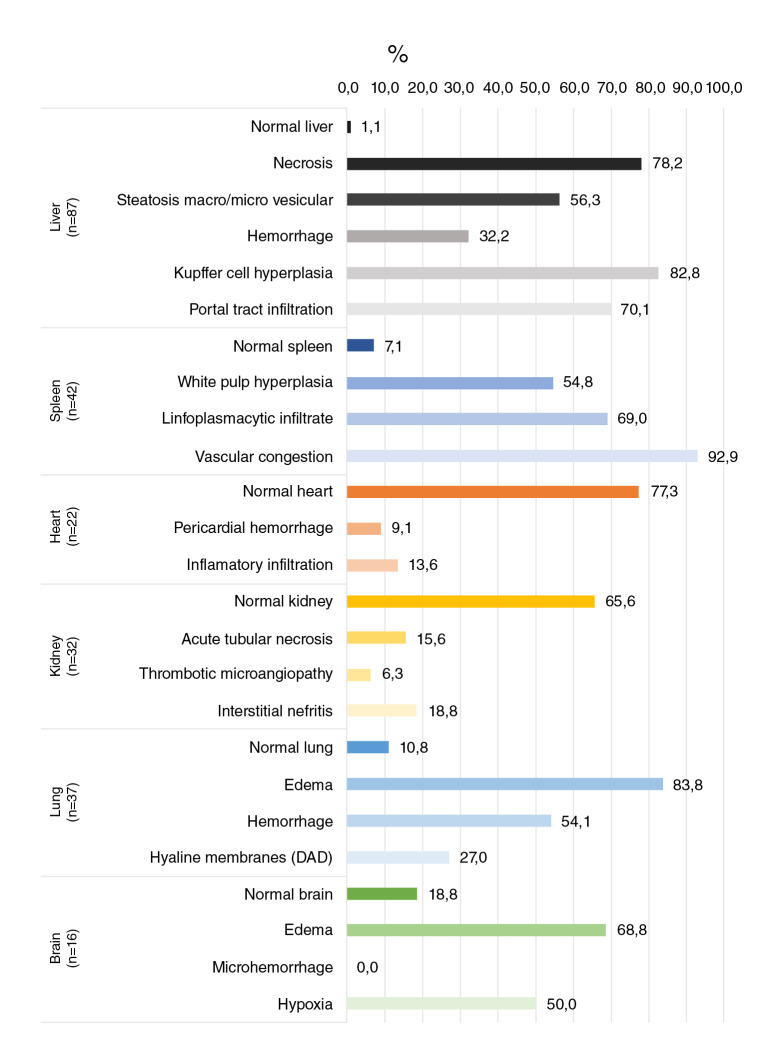
Percentage frequencies of different abnormalities evaluated in tissues from fatal cases

## References

[1] Bhatt S, Gething PW, Brady OJ, Messina JP, Farlow AW, Moyes CL (2013). The global distribution and burden of dengue. Nature.

[2] Stanaway JD, Shepard DS, Undurraga EA, Halasa YA, Coffeng LE, Brady OJ (2016). The global burden of dengue: An analysis from the Global Burden of Disease Study 2013. Lancet Infect Dis.

[3] World Health Organization (2019). Dengue and severe dengue 2018.

[4] Instituto Nacional de Salud (2010). Boletín epidemiológico semanal. Reporte No. semana 52.

[5] Villar LÁ, Gélvez RM, Rodríguez JA, Salgado D, Parra B, Osorio L (2013). Biomarkers for the prognosis of severe dengue. Biomédica.

[6] Yacoub S, Mongkolsapaya J, Screaton G (2016). Recent advances in understanding dengue.

[7] Rojas EM, Herrera VM, Miranda MC, Rojas DP, Gómez AM, Pallares C (2019). Clinical indicators of fatal dengue in two endemic areas of Colombia: A hospital-based case-control study. Am J Trop Med Hyg.

[8] Salgado DM, Eltit JM, Mansfield K, Panqueba C, Castro D, Vega MR (2010). Heart and skeletal muscle are targets of dengue virus infection. Pediatr Infect Dis J.

[9] Jessie K, Fong MY, Devi S, Lam SK, Wong KT (2004). Localization of dengue virus in naturally infected human tissues by immunohistochemistry and in situ hybridization. J Infect Dis.

[10] Nascimento D, Castro AR, Froes IB, Bigaton G, Oliveira EC, Dal Fabbro MF (2011). Clinical and laboratory findings in patients with dengue associated with hepatopathy. Rev Soc Bras Med Trop.

[11] Seneviratne SL, Malavige GN, de Silva HJ (2006). Pathogenesis of liver involvement during dengue viral infections. Trans R Soc Trop Med Hyg.

[12] Ling LM, Wilder-Smith A, Leo YS (2007). Fulminant hepatitis in dengue haemorrhagic fever. J Clin Virol.

[13] Huerre MR, Lan NT, Marianneau P, Hue NB, Khun H, Hung NT (2001). Liver histopathology and biological correlates in five cases of fatal dengue fever in Vietnamese children. Virchows Arch.

[14] Couvelard A, Marianneau P, Bedel C, Drouet MT, Vachon F, Henin D (1999). Report of a fatal case of dengue infection with hepatitis: Demonstration of dengue antigens in hepatocytes and liver apoptosis. Hum Pathol.

[15] Basilio-de-Oliveira CA, Aguiar GR, Baldanza MS, Barth OM, Eyer-Silva WA, Paes MV (2005). Pathologic study of a fatal case of dengue-3 virus infection in Rio de Janeiro, Brazil. Braz J Infect Dis.

[16] Bhamarapravati N, Tuchinda P, Boonyapaknavik V (1967). Pathology of Thailand haemorrhagic fever: A study of 100 autopsy cases. Ann Trop Med Parasitol.

[17] Sarmiento L, Rengifo AC, Rivera J, Neira M, Parra E, Mendez J (2013). Glucógeno hepático en dengue severo: análisis histopatológico. Infectio.

[18] Rivera J, Neira M, Parra E, Méndez J, Sarmiento L, Caldas ML (2014). Detección de antígenos del virus dengue en tejidos post mortem. Biomédica.

[19] Setlik RF, Ouellette D, Morgan J, McAllister CK, Dorsey D, Agan BK (2004). Pulmonary hemorrhage syndrome associated with an autochthonous case of dengue hemorrhagic fever. South Med J.

[20] Gulati S, Maheshwari A (2007). Atypical manifestations of dengue. Trop Med Int Health.

[21] Kuo MC, Lu PL, Chang JM, Lin MY, Tsai JJ, Chen YH (2008). Impact of renal failure on the outcome of dengue viral infection. Clin J Am Soc Nephrol.

[22] Povoa TF, Alves AM, Oliveira CA, Nuovo GJ, Chagas VL, Paes MV (2014). The pathology of severe dengue in multiple organs of human fatal cases: Histopathology, ultrastructure and virus replication. PLoS One.

[23] de Macedo FC, Nicol AF, Cooper LD, Yearsley M, Pires AR, Nuovo GJ (2006). Histologic, viral, and molecular correlates of dengue fever infection of the liver using highly sensitive immunohistochemistry. Diagn Mol Pathol.

